# KREX2 Is Not Essential for Either Procyclic or Bloodstream Form *Trypanosoma brucei*

**DOI:** 10.1371/journal.pone.0033405

**Published:** 2012-03-15

**Authors:** Jason Carnes, Nancy Lewis Ernst, Carey Wickham, Brian Panicucci, Kenneth Stuart

**Affiliations:** 1 Seattle Biomedical Research Institute, Seattle, Washington, United States of America; 2 Department of Global Health, University of Washington, Seattle, Washington, United States of America; University of Texas-Houston Medical School, United States of America

## Abstract

**Background:**

Most mitochondrial mRNAs in *Trypanosoma brucei* require RNA editing for maturation and translation. The edited RNAs primarily encode proteins of the oxidative phosphorylation system. These parasites undergo extensive changes in energy metabolism between the insect and bloodstream stages which are mirrored by alterations in RNA editing. Two U-specific exonucleases, KREX1 and KREX2, are both present in protein complexes (editosomes) that catalyze RNA editing but the relative roles of each protein are not known.

**Methodology/Principal Findings:**

The requirement for KREX2 for RNA editing *in vivo* was assessed in both procyclic (insect) and bloodstream form parasites by methods that use homologous recombination for gene elimination. These studies resulted in null mutant cells in which both alleles were eliminated. The viability of these cells demonstrates that KREX2 is not essential in either life cycle stage, despite certain defects in RNA editing *in vivo*. Furthermore, editosomes isolated from KREX2 null cells require KREX1 for *in vitro* U-specific exonuclease activity.

**Conclusions:**

KREX2 is a U-specific exonuclease that is dispensable for RNA editing *in vivo* in *T. brucei* BFs and PFs. This result suggests that the U deletion activity, which is required for RNA editing, is primarily mediated *in vivo* by KREX1 which is normally found associated with only one type of editosome. The retention of the KREX2 gene implies a non-essential role or a role that is essential in other life cycle stages or conditions.

## Introduction

The mitochondrial genome of *Trypanosoma brucei* encodes 12 genes whose mRNAs undergo post-transcriptional editing that dramatically changes their protein coding sequences [1]–[3]. Using information provided by guide RNA (gRNA) templates, uridine (U) nucleotides are either inserted or deleted at specific editing sites within these RNAs. The extent of editing varies between RNAs, with some RNAs undergoing insertion and deletion of hundreds and tens of Us, respectively. Multiple editing sites are specified by a single gRNA and multiple gRNAs are used in the editing of most mRNAs. This RNA editing is catalyzed by protein complexes called editosomes that contain endoribonuclease, 3′ Terminal Uridylyl-Transferase (TUTase), 3′ U-specific exoribonuclease (exoUase), and RNA ligase activities.

Three compositionally distinct ∼20S editosomes have been identified, each containing a common set of 12 proteins, and a mutually exclusive set of 2 or 3 proteins typified by one of three kinetoplastid RNA editing endonucleases: KREN1, KREN2, or KREN3 [4]–[6]. KREN1 editosomes exclusively contain KREPB8 and exoUase KREX1; KREN2 editosomes exclusively contain KREPB7; KREN3 editosomes exclusively contain KREPB6. In addition, these ∼20S editosomes contain a common set of proteins that includes the heterotrimeric [7] insertion subcomplex (KREPA1, KRET2, and KREL2), the heterotrimeric deletion subcomplex (KREPA2, KREX2, and KREL1), as well as KREPA3, KREPA4, KREPA5, KREPA6, KREPB4, and KREPB5 [8]. Of the two exoUases KREX1 is only in KREN1 editosomes while KREX2 is in the deletion subcomplex of all three editosomes.

Two components of the ∼20S editosome have been shown to have U-specific exoribonuclease activity: KREX1 and KREX2 [9]–[11]. A third editosome protein, KREPA3, was also reported to possess U-specific exoribonuclease activity [12]–[14] but it contains no recognizable catalytic motif and deletion editing activity persists after KREPA3 knockdown [15], [16]. Thus, whether KREPA3 performs such a role *in vivo* is unresolved. RNAi-mediated knockdown of KREX2 produced no defect in either growth or editing, but prevented normal association of KREL1 and KREPA2 with the ∼20S editosomes. In contrast, knockdown of KREX1 resulted in defects in both growth and editing, and prevented normal association of KREN1 with the ∼20S editosomes. Simultaneous RNAi knockdown of both KREX1 and KREX2 produced greater defects in both growth and editing than observed by knockdown of KREX1 alone, suggesting that KREX2 can play a role in RNA editing *in vivo*. In *T. brucei*, both KREX1 and KREX2 have a C-terminal endonuclease/exonuclease/phosphatase (EEP) domain (Pfam 03372) with characteristic conserved catalytic amino acids that indicate that these exonucleases likely have the same catalytic mechanism [17], [18]. Notably, the *Leishmania tarentolae* KREX2 lacks the EEP domain and does not have exonuclease activity [10]. Thus, the nature of KREX2 function in RNA editing remains unclear.

RNAi is a useful albeit unpredictable tool in *T. brucei*. We have shown that RNAi targeting another editosome protein (KREN3) produces an insufficient knockdown to reveal its essentiality; something subsequently demonstrated using the conditional double knockout approach [4], [19]. To unambiguously determine whether KREX2 is an essential gene, we eliminated both endogenous KREX2 gene coding sequences by homologous recombination. We show here using these null mutants that the KREX2 gene is dispensable in both bloodstream and procyclic form *T. brucei*. Procyclic KREX2 null cells exhibit defects in growth, RNA editing, and editosome sedimentation on glycerol gradients. Bloodstream form KREX2 null cells exhibit normal growth, but have defects in RNA editing *in vivo* as well as editosome sedimentation on glycerol gradients. Curiously, only a subset of the phenotypes observed in KREX2 null cells are rescued by reintroduction of an ectopic KREX2 allele. Purification of editosomes from KREX2 null cells using TAP-tag fused to either KREN1 or KREN2 reveals that only editosomes with KREX1 retain exoUase activity *in vitro*. These results demonstrate that KREX1 can be sufficient for editing and perhaps functionally compensate for the loss of KREX2. These results also suggest that most editing exoUase activity *in vivo* is catalyzed by KREX1 and KREX2 has a limited function.

## Results

### Creation of KREX2 null cells

**Figure 1:**
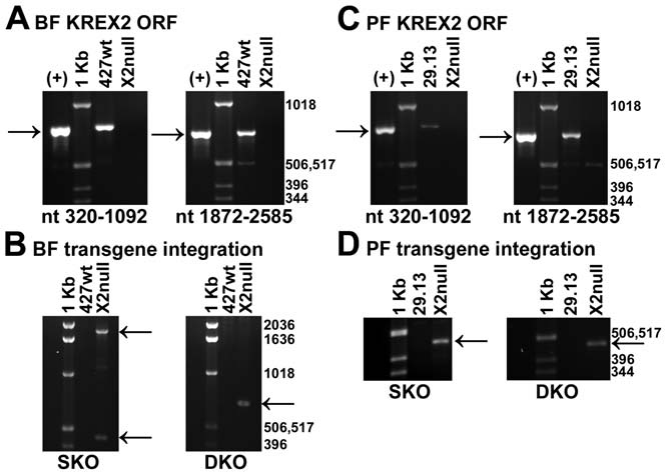
PCR analysis of KREX2 locus demonstrates loss of KREX2 coding sequence in both BF and PF KREX2 null cells. KREX2 coding sequence (marked by arrows) is absent in both BF (**A.**) and PF (**C.**) KREX2 null cells (X2null), but present in parental cells (427wt or 29.13, respectively). Two different primer pairs were used to amplify either nucleotides (nt) 320–1092 (left panel) or nt 1872–2585 (right panel) of KREX2 coding sequence. Positive control (+) is a plasmid containing KREX2 coding sequence. 1 Kb ladder is used as a size reference. Expected integration of knockout constructs in the KREX2 locus was demonstrated in BF (**B.**) and PF (**D.**) KREX2 null cells, but in not parental cells (427wt or 29.13, respectively). Two different primer pairs were used to amplify sequence created by intended integration of first knockout construct (SKO, left panel) or second knockout construct (DKO, right panel) in KREX2 null cells. Arrows mark expected PCR products. For BF SKO PCR, one primer anneals in 2 spots, thereby generating 2 bands.

To create cell lines without KREX2, the endogenous KREX2 alleles were eliminated by homologous recombination in both bloodstream (BF) and procyclic form (PF) cells. PCR analyses detect the KREX2 open reading frame in genomic DNA isolated from parental cells, BF 427 wild-type (wt) or PF 29.13, but not in derived BF-KREX2-null and PF-KREX2-null cell lines (Figure 1). In complementary PCR analyses, products corresponding to the junction of the transgenic knockout constructs in the KREX2 locus are detected in BF-KREX2-null and PF-KREX2-null but not parental cells. The elimination of KREX2 is also demonstrated by Southern analysis of BF-KREX2-null cells and Western analysis of PF-KREX2-null cells (Figure S1).

### Growth of KREX2 null cells

**Figure 2:**
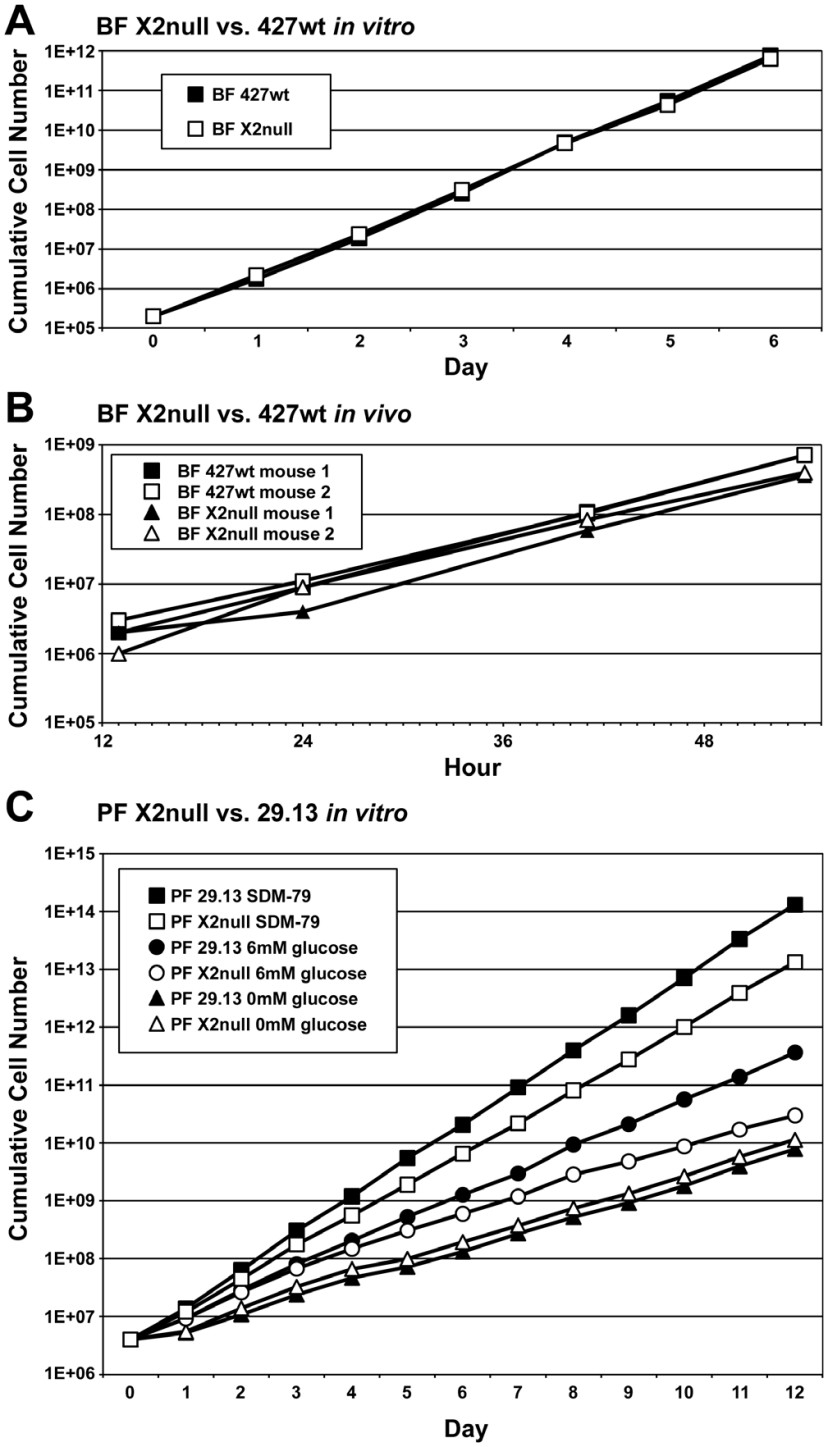
PF KREX2 null cells grow slower than parental cells in vitro, but BF KREX2 null cells grow indistinguishably from parental cells both in vitro and in vivo. **A.** Cumulative growth of BF 427wt (solid squares) and derived KREX2 null cells (open squares) in vitro. **B.** Increasing parasitemia in 2 mice infected with either BF 427wt (squares) or derived KREX2 null cells (triangles) in vivo. **C.** Cumulative growth of PF 29.13 (solid symbols) and derived KREX2 null cells (open symbols) in vitro. In normal SDM-79 media (squares), KREX2 null cells grow more slowly than parental 29.13 cells. In media lacking glucose (triangles) KREX2 null cells grow indistinguishably from parental 29.13 cells. Addition of 6 mM glucose to glucose-free media re-establishes growth defect of KREX2 null cells (circles).

Both BF and PF cells grow in the absence of KREX2 expression (Figure 2). Growth of BF-KREX2-null cells is indistinguishable from parental 427wt cells in both *in vitro* culture and in mice. Growth of PF-KREX2-null cells is slightly slower than parental 29.13 cells in SDM-79 media; however, there is no difference in growth between these cell lines in the absence of glucose. Growth after addition of glucose to glucose-free media demonstrates that the presence of glucose is responsible for the slower growth phenotype of PF-KREX2-null cells. Curiously, growth of the PF-KREX2-rDKO cell line (PF-KREX2-null cells transformed by addition of tetracycline-regulated expression of an ectopic KREX2 allele) is unaltered by tetracycline-induced expression of an ectopic KREX2 allele, despite evidence that the KREX2 protein is produced (data not shown, Figure S1).

### Editing in vivo

**Figure 3:**
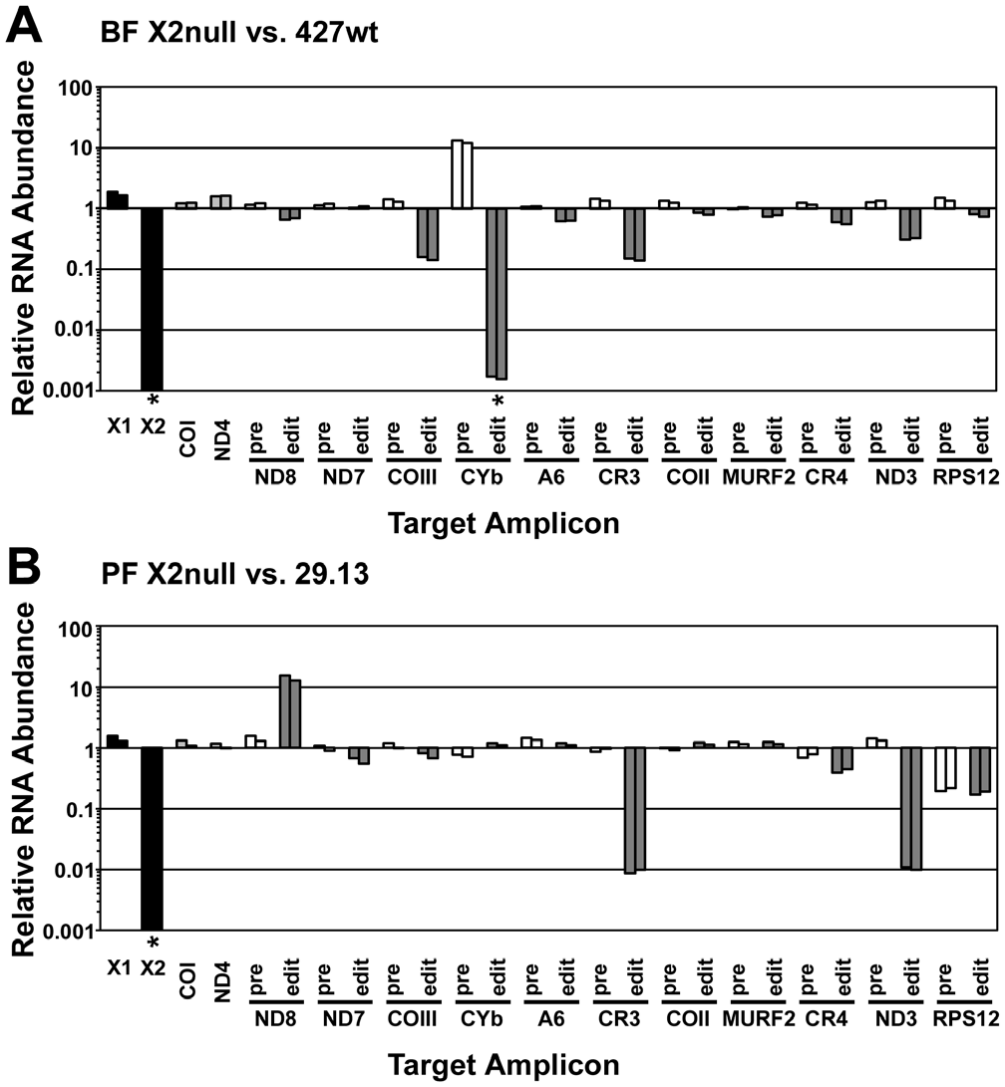
Real-time PCR analysis of KREX2 null cells. RNA abundance for nuclear mRNAs KREX1 and KREX2 (black bars), never-edited mRNAs COI and ND4 (light grey bars), pre-edited mRNAs (white bars), and edited mRNAs (dark grey bars) is calculated relative to parental cell line for both BF (427wt) and PF (29.13). Analysis was performed in triplicate. For each target amplicon, the relative change in RNA abundance was determined by using either 18S rRNA (left bar in each pair) or β-tubulin (right bar in each pair) as an internal control. Asterisks denote mRNAs that were not detected in KREX2 null cells. **A.** A significant loss of CYb editing is shown in BF KREX2 null cells. **B.** PF KREX2 null cells have predominant decreases in CR3 and ND3 edited mRNAs.

The effect of the loss of KREX2 on editing *in vivo* was assessed by quantitative real-time PCR (Figure 3). Comparisons of mRNAs isolated from BF-KREX2-null and parental BF 427wt cells revealed that both KREX2 mRNA and edited CYb mRNA were essentially absent (i.e. not detected) while pre-edited CYb mRNA accumulated in null cells relative to wild type (Figure 3A). The abundance of edited COIII and CR3 transcripts was significantly reduced in null cells relative to wild type, while edited ND3 was reduced to a lesser extent. Other never-edited, edited and pre-edited transcripts were similar in abundance in BF-KREX2-null and wild type cells. Comparisons of mRNAs from PF-KREX2-null and parental 29.13 cells revealed a profile that was distinct from BF-KREX2-null cells (Figure 3B). Again, KREX2 mRNA was not detected in PF-KREX2-null cells. However, the relative abundance of edited CYb and COIII mRNAs was essentially the same in null and wild type cells, while edited CR3, edited ND3, and both edited and pre-edited RPS12 had substantially reduced relative abundance in the null cells, and edited ND8 was increased. As with BF-KREX2-null cells, the levels of other never-edited, edited and pre-edited transcripts are essentially the same in PF-KREX2-null and wild type cells. KREX1 mRNA abundance in both BF and PF KREX2 null cells is essentially the same as in wild type cells. Expression of ectopic KREX2 in the BF-KREX2-rDKO cell line resulted in edited COIII mRNA levels at normal BF 427wt levels, but the relative amount of CYb edited mRNA remained unaltered from that in null cells; KREX2 expression in PF-KREX2-rDKO cells also did not return the amount of edited ND3 mRNA to wild type levels (data not shown).

### Editosome structure

**Figure 4:**
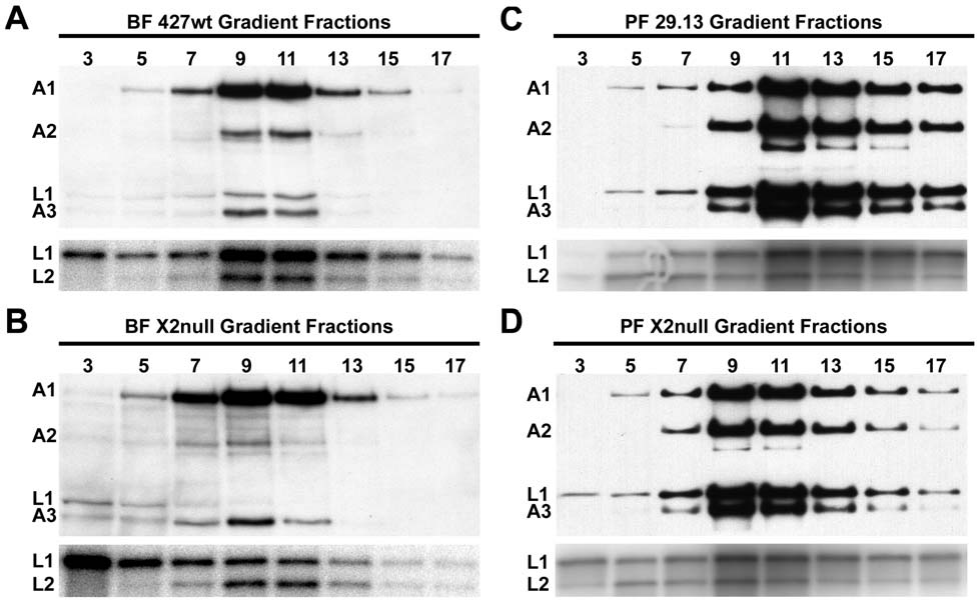
Western and adenylation analyses of glycerol gradient fractionated editosomes from KREX2 null and parental cell lines. Gradient fractions from BF 427wt (**A.**), BF-KREX2-null (**B.**), PF 29.13 (**C.**) and PF-KREX2-null (**D.**) were probed using antibodies recognizing editosome proteins KREPA1, KREPA2, KREL1, and KREPA3 (top panel) or by adenylation of ligases KREL1 and KREL2 (bottom panel). Editosome sedimentation of KREX2 null samples is shifted toward upper part of the gradient (i.e. smaller in size) relative to parental controls. KREL1 particularly shifts up in the gradient.

Editosomes from KREX2 null cells are shifted to lower S values on glycerol gradients compared to parental cells (Figure 4). Western analysis shows that ∼20S editosomes isolated from BF 427wt peak in fractions 9–11, and that editosomes isolated from BF-KREX2-null cells are shifted towards fraction 9. The most obvious evidence of this shift is the increase in KREL1 at the top of the gradient, consistent with its disrupted association with the deletion subcomplex. KREL1 is much more prominent in fraction 3 in samples from KREX2-null cells, as observed by both Western blot and adenylation assay. A more significant shift is observed with editosomes isolated from PF-KREX2-null cells. The ∼20S editosome peaks in fraction 11 in control 29.13 cells, and shifts to fraction 9 in PF cells lacking KREX2. As with BF-KREX2-null cells, PF-KREX2-null cells have a notable increase in KREL1 at the top of the gradient.

### In vitro editing activities in KREX2 null cells

**Figure 5:**
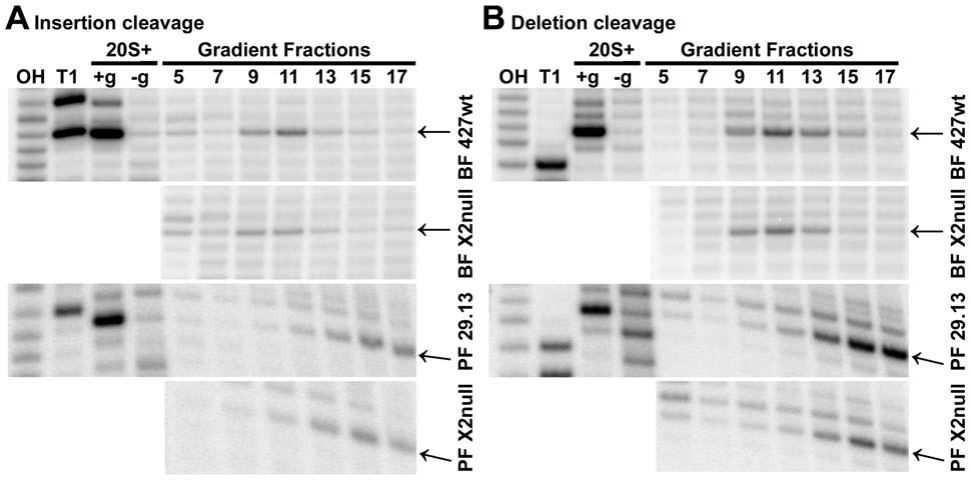
Cleavage activity maintained in KREX2 null cells. Glycerol gradient fractionated editosomes from KREX2 null and parental cell lines were examined for insertion (**A.**) or deletion (**B.**) cleavage activity. Hydroxyl (OH) and T1 nuclease (T1) ladders were used as references. Positive control reaction using 20S mitochondrial fraction (20S+) requires gRNA (+g) for cleavage, which is absent without gRNA (−g). Arrows denote cleavage product.

**Figure 6:**
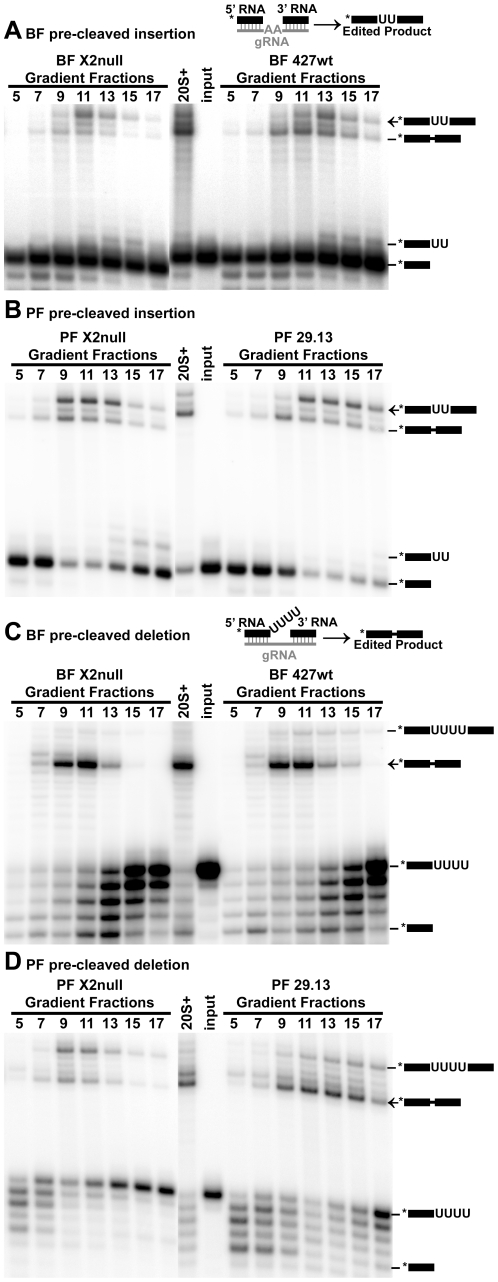
Pre-cleaved editing assays of glycerol gradient fractionated editosomes from KREX2 null and parental cell lines. Editing activities are maintained in KREX2 null cells. 20S glycerol gradient fraction from purified mitochondria is used as a positive control. Asterisks in schematics denote location of radiolabel. Pre-cleaved insertion assays for BF (**A.**) and PF (**B.**) demonstrate KREX2 null cells maintain both TUTase and ligase activity. Pre-cleaved deletion assays for BF (**C.**) and PF (**D.**) demonstrate KREX2 null cells maintain both exoUase and ligase activity.

*In vitro* editing activities are observed in gradient fractions isolated from both KREX2 null and parental cell lines (Figures 5 and 6). KREX2 null cells maintain the ability to cleave both insertion and deletion editing site substrates *in vitro* (Figure 5). While cleavage activity is restricted to the ∼20S peak in fractions from BF cells, activity extends from ∼20S to higher S values in PF, presumably due to the larger amount of editosome isolated. For fractions from both BF and PF KREX2 null cells, the amount of cleavage activity appears slightly reduced compared to control fractions. The observed cleavage activities are present in the same fractions for control parental and derived KREX2 null cells. KREX2 null cells also maintain U addition, U removal, and ligase activities as measured by *in vitro* pre-cleaved editing assays (Figure 6). In contrast to cleavage activities, pre-cleaved editing peaks in different fractions in KREX2 null cells, mirroring the observed shift in editosome proteins towards lower S values. For both BF and PF control fractions pre-cleaved insertion editing peaks at fraction 13, while KREX2 null editing peaks in fraction 11 (Figures 6A & B). The shift in pre-cleaved deletion editing for BF-KREX2-null cells is subtle, most notable by the decrease in edited product and U-deletion intermediates in fraction 15 compared to parental control (Figure 6C). For PF-KREX2-null cells, the peak of pre-cleaved deletion is shifted to fractions 9–11 from fraction 13 in control cells, and the overall amount of editing is decreased in the absence of KREX2 (Figure 6D).

### KREX1 removes Us in the absence of KREX2

**Figure 7:**
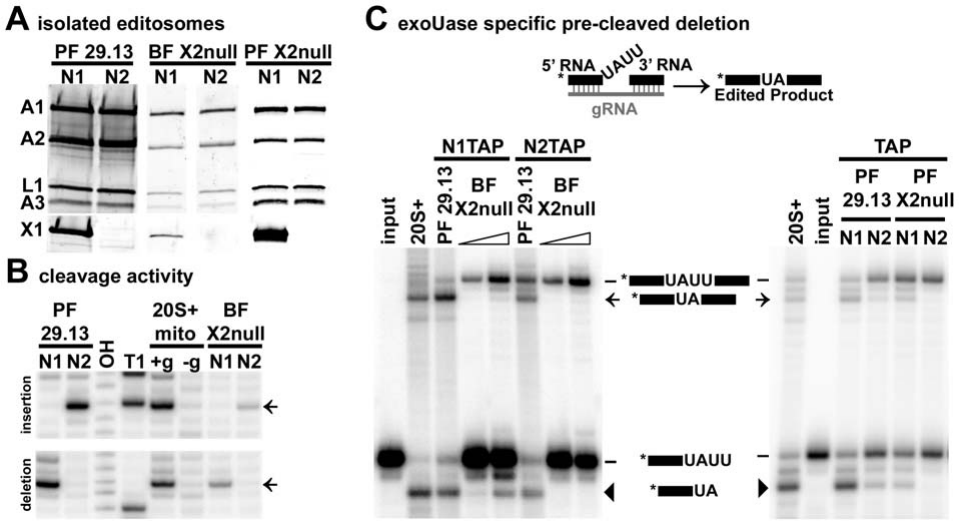
KREN1 editosomes isolated from KREX2 null cells have exoUase activity, while KREN2 editosomes do not. **A.** Western analysis of KREN1 (N1) or KREN2 (N2) TAP purified editosomes from either PF 29.13, BF-KREX2-null, or PF-KREX2-null cells using antibodies recognizing editosome proteins KREPA1, KREPA2, KREL1, and KREPA3 (top panel) or KREX1 (bottom panel). Note KREX1 signal is restricted to N1 editosomes. **B.** Expected cleavage specificity observed with KREN1 and KREN2 editosomes isolated from BF-KREX2-null cells. Insertion (top panel) and deletion (bottom panel) cleavage labeled as in Figure 5. **C.** Modified pre-cleaved deletion substrate assays U-specific exonuclease activity. Schematic depicts base change from U to A within the unpaired string of Us typically removed in pre-cleaved deletion assays. Left panel shows that only KREN1 editosomes from BF-KREX2-null cells have U-specific exonuclease activity, while it is possessed by both KREN1 and KREN2 editosomes from PF 29.13. Right panel shows similar restriction of U-specific exonuclease activity to KREN1 editosomes from PF-KREX2-null cells. Open triangles indicate increasing amount of extract used in assays. Arrow denotes fully edited product. Note also the lack of -2U product (indicated by black wedges) with KREN2 editosomes isolated from KREX2 null cells.

*In vitro* U deletion activity is restricted to editosomes that contain KREX1 in KREX2 null cells (Figure 7). While KREX2 and KREPA3 are present in all types of editosomes, KREX1 is restricted to KREN1 editosomes. We therefore isolated KREN1 and KREN2 editosomes from KREX2 null cells to determine whether U removal is performed solely by KREX1 in the absence of KREX2. As expected, KREX1 is found in KREN1 but not KREN2 editosomes (Figure 7A). The cleavage activity of these editosomes confirms the expected specificity and functional capacity of these samples (Figure 7B). To examine U-specific exonuclease activity of the isolated KREN1 and KREN2 editosomes, samples were tested using a modified pre-cleaved deletion substrate with a single U replaced by an A (Figure 7C, top). U-specific exonuclease activity is observed with KREN1 editosomes, but not with KREN2 editosomes in KREX2 null cells from both BF and PF (Figure 7C, left and right, respectively). In contrast, both KREN1 and KREN2 editosomes show U-specific deletion when isolated from PF 29.13 cells. The absence of the -2U intermediate product is particularly noticeable for KREN2 editosomes isolated from KREX2 null cells.

## Discussion

The data we present here demonstrate that KREX2 is not required for RNA editing, suggesting that U deletion *in vivo* is primarily mediated by the subset of editosomes that contain KREX1. *In vitro* deletion assays reveal that KREX1 is necessary for the U-specific exonuclease activity of editosomes isolated from KREX2 null cells. The loss of KREX2 leads to a smaller editosome size, as determined by glycerol gradient sedimentation, and a disruption to the deletion subcomplex, which is apparent by the shift of KREL1 to lower S value fractions. The abundance of most edited, pre-edited, and never edited transcripts is unaltered in KREX2 null cells compared to controls, with distinct exceptions. Edited CYb is essentially eliminated in BF-KREX2-null cells, while edited ND3 and edited CR3 are severely decreased in PF-KREX2-null cells. Regardless of these editing defects, the persistence of cell growth in the absence of KREX2 in both BF and PF stages shows that it is not required catalytically or structurally for editing to occur.

Several lines of evidence indicate that KREX2 has been eliminated from both BF-KREX2-null and PF-KREX2-null cells. First, the open reading frame of KREX2 was not detected by two distinct sets of primers by PCR, while PCR products consistent with the integrated knockout constructs were found (Figure 1). Second, Southern analysis of genomic DNA from BF-KREX2-null cells demonstrates the loss of the KREX2 gene. Third, Western analysis using anti-KREX2 antibody shows that PF-KREX2-null cells lack KREX2 protein. Fourth, real-time PCR analysis shows that KREX2 mRNA is gone in both BF-KREX2-null and PF-KREX2-null cells. Fifth, the shift in editosome sedimentation matches independently derived KREX2 RNAi results. Finally, the loss of U-specific exonuclease activity in editosomes lacking KREX1 shows the loss of activity consistent with KREX2. Together these data show that KREX2 has been eliminated from both BF and PF cells, and thus KREX2 is dispensable for growth in *T. brucei*.

While the growth of BF-KREX2-null cells was indistinguishable from parental control cells both *in vivo* and *in vitro*, PF-KREX2-null cells grew slower than parental 29.13 cells in media containing glucose (Figure 2). Unlike PF cells BF cells are normally dependent on glucose. Ectopic expression of KREX2 in the PF null cells, which was confirmed by Western analysis and resulted in normal editosome sedimentation but did not rescue normal growth in PF (data not shown and Figure S1). A trivial possibility is that the glucose-dependent growth defect in the null cells is coincidental and unrelated to the loss of KREX2. Alternatively, loss of KREX2 may have resulted in anomalies in mitochondrial function such as disrupting the balance between oxidative phosphorylation and glycolysis as implied by the growth deficiency in glucose [20]–[22]. In this case, differences between ectopic and endogenous expression may have resulted in the different growth rates. For example, different rates of protein expression may have altered editosome assembly, turnover or function.

KREX2 mRNA is absent in null mutant cells but the levels of KREX1 mRNA and of most edited transcripts are unaltered compared to parental control cells. However, there are distinct defects in RNA editing *in vivo* in BF and PF KREX2 null cells with effects primarily on Cytochrome mRNAs in BFs and on complex I mRNAs in PFs. The loss of KREX2 appears to result in transcript-specific defects in editing. The lack of edited CYb, increased relative abundance of pre-edited CYb, and ∼85% relative reductions of edited CR3, COIII and ∼70% reduction of edited ND3 mRNA in BF cells is consistent with the normal levels of these edited mRNAs and their protein products in this life cycle stage. In particular, edited CYb mRNA, which only has insertion sites, is normally abundant in PFs and is essentially absent in slender BFs [23]. In previous experiments, edited CYb was not detected in BF cells in which transcription of KREN2 or KREPA3 was expressed using a similar conditional knockout system, suggesting that CYb editing is particularly sensitive to changes to the editing machinery [15], [24]. The rescue in BFs of editing of COIII but not CYb mRNA upon expression of an ectopic KREX2 (data not shown) may also reflect the process that controls the differential editing between life-cycle stages. The substantial decreases in edited ND3 and CR3 mRNAs and large increase in edited ND8 mRNAs in PF-KREX2-null cells relative to the parental cells implies that alterations to complex I are tolerated in this life cycle stage, which is consistent with previous observations [25]. The knockdown of both pre-edited and edited RPS12 suggests that aberrant editing of this transcript increases its turnover rate. The lack of rescue of particular editing defects upon expression of an ectopic KREX2 gene could reflect a change unrelated to loss of the KREX2 gene; however, it seems unlikely that such coincidental defects would specifically disrupt editing of different mRNAs. Unknown quantitative or qualitative differences between KREX2 expressed ectopically and KREX2 expressed from the endogenous locus might compromise its ability to incorporate into editosomes or function at wild type levels.

The shift of editosomes from both BF and PF null mutants to lower S values compared to control parental cell lines is consistent with the loss of KREX2 resulting in structural disruption of the deletion subcomplex (KREX2/KREPA2/KREL1). The greater shift of KREL1 compared to KREPA2 may reflect the association of the latter with KREPA3 and KREPA6 editosome components, and suggests that KREX2 and KREL1 directly interact [8]. RNAi knockdown of KREX2 in PF resulted in a similar shift of KREL1, but not KREPA2, to lower S values [11]. The structural disruption caused by the loss of KREX2 apparently does not prevent KREL1 function, since KREL1 is essential [26]. Whether KREL1 can perform its essential function in *trans* is unknown.

KREX2 null cells maintain the *in vitro* editing activities present in parental control cells, but assays of glycerol gradient fractions reveal clues concerning both KREX2 and editosome function. While pre-cleaved editing activities are present in KREX2 null extracts, they are uniformly shifted up toward smaller S values, mimicking the profile of editosome proteins observed by Western blot (Figures 4 and 6). In contrast, cleavage activity is observed in the same fractions from both KREX2 null and parental cells, namely ∼20S and higher (Figure 5). These results suggest that cleavage activity requires more intact editosomes, i.e. interactions among multiple proteins, while pre-cleaved activities can be performed by smaller partial complexes. The somewhat diminished editing activities in KREX2 null extracts compared to parental cells appears largely due to decreased post-cleavage activities and is consistent with the loss of KREX2 U removal activity. Thus KREX2 has exonuclease activity *in vitro*, but whether it functions as an exonuclease *in vivo* is unknown given the functional redundancy of KREX1. Nevertheless, in the absence of KREX2 U removal *in vitro* is restricted to editosomes that contain KREX1 (Figure 7). The presence of KREX1 in editosomes with deletion site cleavage specificity, combined with the dispensability of KREX2, suggests that U removal *in vivo* is primarily done by KREX1.

What role might KREX2 play in RNA editing *in vivo*? It could be an evolutionary relic whose role in U removal activity has been supplanted by KREX1. The smaller *Leishmania* KREX2 is consistent with this possibility, as it lacks the catalytic domain and hence exoUase activity. KREX2 may have a role in the structural architecture of the editosome. While this structural role may not be essential, at least in the laboratory, it may influence subtle aspects of editosome function such as differential editing during the life cycle. For example, KREX2 may influence the interactions of accessory factors with the editosome (Lerch, et al unpublished). It might also provide a U removal activity in specific circumstances such as for unusual editing sites from which numerous Us are removed, or a proofreading activity that removes excess Us that have been added in error. Hence, KREX2 absence may only be deleterious under conditions where a substantial decrease in editing fidelity cannot be compensated.

## Materials and Methods

### Ethics Statement

Trypanosome growth in mice was assessed with the approval of the Institutional Animal Care and Use Committee at Seattle Biomedical Research Institute under protocol KS-01. Generation of transgenic *T. brucei* cell lines was performed with the approval of the Institutional Biosafety Committee at Seattle Biomedical Research Institute under application R1021.

### PCR analysis of genomic DNA

PCR was used to determine if the KREX2 coding sequence had been eliminated by the intended homologous recombinations. Primer sequences are in Table S1. Two sets of primers were used to detect two distinct portions of the KREX2 coding sequence; primers X2for4571 and X2rev3135 amplify nucleotides 320–1092 (773 bp product), while primers X2for3127 and X2rev3177 amplify nucleotides 1872–2585 (714 bp product). Primers that flank the junction between the inserted knockout construct and the genomic region 3′ of KREX2 were used to amplify products that demonstrate the intended homologous recombination. Primers pLEW13for5154 (anneals to two places in pLEW13 insert) and X2rev3utr5952 generate products of 473 bp and 1811 bp that indicate integration of the first BF knockout construct. Primers pLEW90for5209 and X2rev3utr5952 generate a product of 702 bp that indicates integration of the second BF knockout construct. Primers BSDfor7258 and X2rev3utr5952 generate a product of 468 bp that indicates integration of the first PF knockout construct. Primers PACfor3748 and X2rev3utr5952 generate a product of 456 bp that indicates integration of the second PF knockout construct.

### Plasmid constructs

Plasmids pSKO-KREX2 (single knockout) and pDKO-KREX2 (double knockout) were created using published methods to eliminate both KREX2 alleles in BF cells [26], [27], while plasmids pBSD-KREX2 (single knockout) and pPAC-KREX2 (double knockout) were created using published methods to eliminate both KREX2 alleles in PF cells [27]–[29]. To generate pSKO-KREX2, the 5′ and 3′ UTRs of KREX2 were PCR amplified from 427 genomic DNA using 5FOR-3312, 5REV-3313, 3FOR-3279, and 3REV-3280 primers. The 313 bp KREX2 5′ UTR and 344 bp KREX2 3′ UTR PCR products were cloned into the NotI/MluI and StuI/XbaI sites of pLew13, respectively creating pSKO-KREX2. pDKO-KREX2 was created by replacing the SwaI/XhoI fragment (containing Neo^r^ marker) of pSKO-KREX2 with the 2491 bp StuI/XhoI fragment (containing Hyg^r^ marker) from pLew90.

To generate plasmids pBSD-KREX2 and pPAC-KREX2, the 5′ and 3′ UTRs of KREX2 were PCR amplified from 427 genomic DNA using 5FOR-6519, 5REV-6664, 3FOR-6521, and 3REV-6522 primers. The two PCR products containing 423 bp KREX2 5′ UTR and 344 bp KREX2 3′ UTR were then joined by another PCR amplification to create a single PCR product flanked by *Not*I sites and a *Hin*dIII, *Pme*I, and *Bam*HI cloning site between the 5′ and 3′ UTRs, as previously described [28], [29]. This PCR product was cloned into the *Not*I site of pGEM-5Zf(+) vector (Promega), and the blasticidin (BSD) or puromycin (PAC) resistance marker (from knockout plasmids targeting *TbGPI12* [30] and *GPIdeAc* [28], respectively) was then inserted between the UTRs using the *Hin*dIII and *Bam*HI sites to create plasmids pBSD-KREX2 and pPAC-KREX2.

Wild-type KREX2 gene was cloned into the pLEW79 plasmid [27], creating the pReg-KREX2 plasmid as follows. A 2746 bp PCR product containing the KREX2 open reading frame was PCR amplified from 427 genomic DNA with *Pfu* polymerase using 5orf-3281 and 3orf-3309 primers. The KREX2 ORF was cloned into the *Hin*dIII/*Bam*HI sites of pLEW79. This plasmid was used to create cell lines with tetracycline-induced expression of an ectopic KREX2 allele from the rDNA locus.

### Cell lines

BF-KREX2-null cell line was generated by a series of transfections to introduce the pSKO-KREX2 and pDKO-KREX2 plasmids sequentially into 427 strain cells. First, BF427 cells were transfected with 10 µg *Not*I-linearized pSKO-KREX2, and recombinants were selected by G418 resistance. The second endogenous KREX2 allele was eliminated by transfection with 10 µg *Not*I-linearized pDKO-KREX2 and subsequent hygromycin selection. Cells were grown in HMI-9 media containing 2.5 µg/ml G418 and 5 µg/ml hygromycin. PF-KREX2-null cell line was generated by a series of transfections to introduce the pBSD-KREX2 and pPAC-KREX2 plasmids sequentially into PF 29.13 strain cells [27], [31]. First, 29.13 cells were transfected with 10 µg *Not*I-linearized pBSD-KREX2, and recombinants were selected by blasticidin resistance. The second endogenous KREX2 allele was eliminated by transfection with 10 µg *Not*I-linearized pPAC-KREX2 and subsequent puromycin selection. BF-KREX2-null cells were grown in HMI-9 media containing 2.5 µg/ml G418 and 5 µg/ml hygromycin. PF-KREX2-null cells were grown in HMI-9 media containing 15 µg/ml G418, 25 µg/ml hygromycin, 1 µg/ml puromycin, and 10 µg/ml blasticidin. Proper integration of each plasmid was confirmed by PCR in both BF and PF, as well as Southern analysis (BF only; Figure S1). TAP-tagged versions of KREN1 or KREN2 were introduced into both BF and PF KREX2 null cells using plasmids previously described [5]. A regulatable ectopic KREX2 allele was subsequently introduced into the rDNA intergenic locus of both BF-KREX2-null and PF-KREX2-null cells by transfection with *Not*I-linearized pReg-KREX2 and selection in 2.5 µg/ml phleomycin, generating BF-KREX2-rDKO and PF-KREX2-rDKO cell lines, respectively. Induction of pReg-KREX2 used 1 µg/ml tetracycline.

### Growth of cells in vitro

BF cells were grown in HMI-9 with 10% FBS. PF cells were grown in SDM-79 with 10% FBS, or in SDM-79 modified by the removal of glucose and glucosamine with 10% FBS that had been dialyzed to remove glucose; as noted glucose was returned to depleted media at final concentration of 6 mM [32]. For each cell line, cell density was measured by Coulter counter, and subsequently each culture was reseeded at 2×10^5^ cells/mL in 10 mL (BF) or at 1×10^6^ cells/mL in 5 mL (PF).

#### Growth of cells in vivo

For both BF-427wt and BF-KREX2-null cell lines, 2.5×10^7^ total cells from log-phase cultures grown in HMI-9 with 10% FBS were centrifuged at 1300 g for 10 minutes at room temperature, washed once with 20 mL 1× PBS-G, and then resuspended in 1 mL 1× PBS-G so that the 200 µL injection volume contained 5×10^6^ cells. For each cell line, two ∼20 g BALB/c mice were infected via intraperitoneal injection with 5×10^6^ trypanosomes. Parasitemia was measured at various time points by tail prick to draw 2 µl of blood that was preserved in 200 µl of Fixing Solution (3.7% formaldehyde, 1× SSC). The number of trypanosomes was then counted using a hemocytometer. Mice were killed by CO_2_ asphyxiation when their parasitemia neared 1×10^9^ trypanosomes/mL.

### Fractionation of cell lysates on glycerol gradients

Fractionation of BF whole cell lysates on 10–30% glycerol gradients was performed as previously described [24] with the following differences: ∼1.7×10^9^ cells were lysed and fractionated on each gradient, and the gradients were centrifuged at 38,000 rpm in a Beckman SW40 Ti rotor for 8 hours at 4°C. Briefly, cells were resuspended in lysis buffer (10 mM Tris pH 7.2, 10 mM MgCl2, 100 mM KCl, 1 mM Pefabloc, 2 µg/mL leupeptin, 1 µg/ml pepstatin, 1 mM DTT) to final volume of 900 µL, and 100 µL 10% Triton X-100 was added. Samples were mixed by inversion for 15 minutes at 4°C and cleared by two centrifugation steps of 17,000× g for 15 minutes at 4°C. After fractionation, glycerol gradients were divided into 0.5 mL fractions from the top, flash frozen on liquid nitrogen, and stored at −80°C. For each sample within an experiment, equivalent cell numbers were lysed. Fractionation of PF whole cell lysates was done as for bloodform, except ∼4×10^9^ cells were used. Positive control ∼20S samples from purified PF mitochondria (IsTaR 1.7a strain) were generated as previously described [24], [33].

### TAP-tag purifications

Isolation of PF control KREN1 and KREN2 ∼20S editosomes have been previously described [5], [6]. Tandem affinity purification (TAP) was used to isolate KREN1 and KREN2 editosomes from the background of KREX2 null cells that had been induced to express tagged protein by addition of 500 ng/mL tetracycline. Briefly, equivalent cell numbers (∼2.8×10^9^ cells for BF; ∼2×10^10^ cells for PF;) were harvested and lysed in 20 mL of IPP150, 1% Triton X-100, and Complete protease inhibitors (Roche) at 4°C, and then clarified by centrifugation at 10,000× g. Purification of editosomes via TAP-tagged KREN1 or KREN2 used sequential IgG and Calmodulin affinity chromatography as previously described [34].

### Western analyses

Glycerol gradient fractions (30 µL) were separated by electrophoresis on 10% SDS-PAGE gels, transferred to Immobilon-P membranes (Fisher), and probed using monoclonal antibodies against KREPA1, KREPA2, KREL1, and KREPA3 as previously described [35]. Western analysis of TAP-tag isolated complexes in Figure 7A was performed using a Licor Odyssey scanner essentially as previously described [6]. Briefly, samples were resolved on 10% SDS-PAGE gel, transferred to Immobilon-FL membranes (LiCor), and blocked in Odyssey blocking buffer. Blots were simultaneously probed with mouse monoclonal antibodies against KREPA1, KREPA2, KREL1, and KREPA3 as above, and 1∶2,000 rabbit polyclonal antibody against KREX1 [11]. Blots were then probed with 1∶15,000 IRDye680 conjugated goat anti-rabbit (LiCor) and IRDye800 conjugated goat anti-mouse (Rockland) secondary antibodies and visualized on a LiCor Odyssey scanner.

### In vitro enzymatic assays

For standard pre-cleaved editing and endonuclease cleavage assays, 15 or 10 µL of glycerol gradient fractions were used, respectively. Reactions were incubated at 28°C for 3 hours. For all endonuclease and precleaved assays, RNAs were ethanol precipitated, resolved on 11% polyacrylamide 7M urea gels, and analyzed by PhosphorImager (Molecular Dynamics). A6-derived substrate assays follow standard protocols described in detail elsewhere [24], [36]–[38].

#### A6-derived insertion endonuclease

Cleavage of 70 nt A6-eES1 pre-mRNA with gA6[14] gRNA was performed as described [24].

#### A6-derived deletion endonuclease

Cleavage of 73 nt A6short/TAG.1 pre-mRNA with D34 gRNA was performed as described [24].

#### A6-derived pre-cleaved editing

Standard pre-cleaved deletion and insertion editing were assayed as previously described using 5′-labeled U5 5′CL and U5 3′CL with gA6[14]PC-del and 5′-labeled 5′CL18 and 3′CL13pp with gPCA6-2A RNAs, respectively [39], [40]. For assays with pre-cleaved deletion substrate U5 5′CL3A that is modified to test U specificity, either 5 or 10 µL of calmodulin eluate from BF samples or 7 µL from PF samples was assayed as previously described [41].

### Real-time PCR analysis

Real-time PCR was performed as previously described, with values normalized to either 18S rRNA or β-tubulin and an internal control [4], [11], [24]. Primers for CR3 and ND8 targets are in Table S1. Amplicons for these primer sets were sequenced to confirm they amplified the specified target. For each RNA measured, the average of three cycle threshold (C_T_) values was used in calculations. Relative changes in target amplicons were determined by using the Pfaffl method, with PCR efficiencies calculated by linear regression using LinRegPCR [42], [43].

## Supporting Information

Figure S1 **Southern and Western analyses demonstrate elimination of KREX2 in BF-KREX2-null and PF-KREX2-null cells.** **A.** Genomic DNA from parental BF-427wt and derived KREX2 single knockout (SKO) and KREX2-null (X2null) cell lines was subjected to Southern analysis using a radiolabeled probe to detect the KREX2 open reading frame (KREX2 ORF). The band corresponding to KREX2 is present in DNA from either BF-427wt or BF-KREX2-SKO cell lines, but completely absent in DNA from BF-KREX2-null cells. **B.** The same genomic DNAs used in panel A were also analzyed using a radiolabeled probe to detect the 3′ intergenic region of KREX2. Hybridization with this probe permits simultaneous detection of the endogenous KREX2 alleles, the first allele knockout (1^st^ allele KO) with T7 RNA polymerase and Neo^R^, and the second allele knockout (2^nd^ allele KO) with tetracycline regulator and Hyg^R^. While parental BF-427wt cells have only endogenous KREX2, the BF-KREX2-null cells lack endogenous KREX2 and only display hybridization consistent with the knockout constructs that replaced each KREX2 allele. **C.** Western analysis of ∼20S peak glycerol gradient fractions from parental PF 29.13 or PF-KREX2-null (X2null) and derived cells. Anti-KREX2 antibody reveals presence of KREX2 in 29.13 cells, and absence in PF-KREX2-null cells. Expression of the tetracycline (tet) regulatable ectopic KREX2 allele in the PF-KREX2-null+KREX2Reg cell line (X2null+X2reg) was demonstrated in the presence of tet (+tet), but not in its absence (−tet). The sizes of proximate marker bands are as indicated. The amount of KREX2 in extracts from BF cells was below the limit of detection with this antibody (data not shown). ***Southern analysis.*** Genomic DNA was isolated from each cell line (ACS protocol). For each cell line, 20 µg of genomic DNA was digested with either *Eco*RI or a combination of *Bam*HI and *Kpn*I and then fractionated by electrophoresis on 0.8% agarose gel. *Eco*RI digestion of genomic DNA generates a 1141 bp fragment (WT), a 5550 bp fragment (first knockout), and and a 2049 bp fragment (second knockout) detected by *Not*I probe. *Bam*HI and *Kpn*I digestion of genomic DNA generates a 4104 bp fragment (WT) detected by *Eco*RI probe. Two different DNAs were used as templates for making radiolabeled probes: an 823 bp *Eco*RI fragment of pReg-KREX2 plasmid was used to hybridize to the KREX2 open reading frame, and a 358 bp *Xba*I/*Not*I fragment of pSKO-KREX2 plasmid was used to hybridize to the 3′ intergenic region of KREX2. Radiolabeled DNA probes were generated using Ready-To-Go DNA Labeling Beads (GE Healthcare) and α^32^P dCTP according to manufacturer's protocol. After labeling, DNA probes were purified using MicroSpin G-25 spin columns (GE Healthcare) according to manufacturer's protocol to remove unincorporated dCTP. ***KREX2 Western analysis.*** For each sample, 30 µL of glycerol gradient fraction 11, corresponding to ∼20S peak, was separated by electrophoresis on TGX 10% SDS-PAGE gels (BioRad), transferred to Immobilon-P membranes (Fisher), and probed using 1∶5 diluted affinity-purified rabbit polyclonal antibody against KREX2 as previously described [11]. Blot was then probed with 1∶2000 goat anti-rabbit conjugated HRP secondary antibody and visualized by chemiluminescence (Pierce) detected by x-ray film. PageRuler ladder (Fermentas) was used as a size marker.
(TIF)

Table S1 **Primer sequences used in this study.**
(XLS)

## References

[1] Stuart KD, Schnaufer A, Ernst NL, Panigrahi AK (2005). Complex management: RNA editing in trypanosomes.. Trends Biochem Sci.

[2] Aphasizhev R, Aphasizheva I (2011). Mitochondrial RNA processing in trypanosomes.. Res Microbiol.

[3] Hajduk S, Ochsenreiter T (2010). RNA editing in kinetoplastids.. RNA Biol.

[4] Carnes J, Trotter JR, Peltan A, Fleck M, Stuart K (2008). RNA editing in *Trypanosoma brucei* requires three different editosomes.. Mol Cell Biol.

[5] Panigrahi AK, Ernst NL, Domingo GJ, Fleck M, Salavati R (2006). Compositionally and Functionally Distinct Editosomes in *Trypanosoma brucei*.. RNA.

[6] Carnes J, Zelaya-Soares C, Wickham C, Stuart K (2011). Endonuclease Associations with Three Distinct Editosomes in *Trypanosoma brucei*.. J Biol Chem.

[7] Schnaufer A, Ernst N, O'Rear J, Salavati R, Stuart K (2003). Separate Insertion and Deletion Sub-complexes of the *Trypanosoma brucei* RNA Editing Complex.. Mol Cell.

[8] Schnaufer A, Wu M, Young-jun P, Nakai T, Deng J (2010). A Protein-Protein Interaction Map of Trypanosome ∼20s Editosomes.. J Biol Chem.

[9] Kang X, Rogers K, Gao G, Falick AM, Zhou S (2005). Reconstitution of uridine-deletion precleaved RNA editing with two recombinant enzymes.. Proc Natl Acad Sci U S A.

[10] Rogers K, Gao G, Simpson L (2007). Uridylate-specific 3′ - 5′ Exoribonucleases Involved in Uridylate-deletion RNA Editing in Trypanosomatid Mitochondria.. J Biol Chem.

[11] Ernst NL, Panicucci B, Carnes J, Stuart K (2009). Differential Functions of Two Editosome ExoUases in *Trypanosoma brucei*.. RNA.

[12] Niemann M, Brecht M, Schluter E, Weitzel K, Zacharias M (2008). TbMP42 is a structure-sensitive ribonuclease that likely follows a metal ion catalysis mechanism.. Nucl Acids Res.

[13] Brecht M, Niemann M, Schlüter E, Müller UF, Stuart K (2005). TbMP42, a protein component of the RNA editing complex in African trypanosomes has endo-exoribonuclease activity.. Molecular Cell.

[14] Niemann M, Kaibel H, Schluter E, Weitzel K, Brecht M (2009). Kinetoplastid RNA editing involves a 3′ nucleotidyl phosphatase activity.. Nucleic Acids Res.

[15] Guo X, Ernst NL, Stuart KD (2008). The KREPA3 zinc finger motifs and OB-fold domain are essential for RNA editing and survival of *Trypanosoma brucei*.. Mol Cell Biol.

[16] Law JA, O'Hearn SF, Sollner-Webb B (2008). Trypanosoma brucei RNA editing protein TbMP42 (band VI) is crucial for the endonucleolytic cleavages but not the subsequent steps of U-deletion and U-insertion.. RNA.

[17] Dlakic M (2000). Functionally unrelated signalling proteins contain a fold similar to Mg2+-dependent endonucleases.. Trends Biochem Sci.

[18] Mian IS, Worthey EA, Salavati R (2006). Taking U out with two nucleases?. BMC Bioinformatics.

[19] Trotter JR, Ernst NL, Carnes J, Panicucci B, Stuart K (2005). A Deletion Site Editing Endonuclease in *Trypanosoma brucei*.. Mol Cell.

[20] Bringaud F, Riviere L, Coustou V (2006). Energy metabolism of trypanosomatids: adaptation to available carbon sources.. Mol Biochem Parasitol.

[21] Coustou V, Biran M, Breton M, Guegan F, Riviere L (2008). Glucose-induced remodeling of intermediary and energy metabolism in procyclic Trypanosoma brucei.. J Biol Chem.

[22] Lamour N, Riviere L, Coustou V, Coombs GH, Barrett MP (2005). Proline metabolism in procyclic Trypanosoma brucei is down-regulated in the presence of glucose.. J Biol Chem.

[23] Feagin JE, Jasmer DP, Stuart K (1987). Developmentally regulated addition of nucleotides within apocytochrome b transcripts in Trypanosoma brucei.. Cell.

[24] Carnes J, Trotter JR, Ernst NL, Steinberg AG, Stuart K (2005). An Essential RNase III Insertion Editing Endonuclease in *Trypanosoma brucei*.. Proc Natl Acad Sci U S A.

[25] Verner Z, Cermakova P, Skodova I, Kriegova E, Horvath A (2010). Complex I (NADH:ubiquinone oxidoreductase) is active in but non-essential for procyclic Trypanosoma brucei.. Mol Biochem Parasitol.

[26] Schnaufer A, Panigrahi AK, Panicucci B, Igo RP, Salavati R (2001). An RNA ligase essential for RNA editing and survival of the bloodstream form of *Trypanosoma brucei*.. Science.

[27] Wirtz E, Leal S, Ochatt C, Cross GAM (1999). A tightly regulated inducible expression system for conditional gene knock-outs and dominant-negative genetics in *Trypanosoma brucei*.. Mol Biochem Parasitol.

[28] Guther ML, Leal S, Morrice NA, Cross GA, Ferguson MA (2001). Purification, cloning and characterization of a GPI inositol deacylase from Trypanosoma brucei.. EMBO J.

[29] Chang T, Milne KG, Guther ML, Smith TK, Ferguson MA (2002). Cloning of Trypanosoma brucei and Leishmania major genes encoding the GlcNAc-phosphatidylinositol de-N-acetylase of glycosylphosphatidylinositol biosynthesis that is essential to the African sleeping sickness parasite.. J Biol Chem.

[30] Guther ML, Lee S, Tetley L, Acosta-Serrano A, Ferguson MA (2006). GPI-anchored proteins and free GPI glycolipids of procyclic form Trypanosoma brucei are nonessential for growth, are required for colonization of the tsetse fly, and are not the only components of the surface coat.. Mol Biol Cell.

[31] Roper JR, Guther ML, Macrae JI, Prescott AR, Hallyburton I (2005). The suppression of galactose metabolism in procylic form Trypanosoma brucei causes cessation of cell growth and alters procyclin glycoprotein structure and copy number.. J Biol Chem.

[32] Furuya T, Kessler P, Jardim A, Schnaufer A, Crudder C (2002). Glucose is toxic to glycosome-deficient trypanosomes.. Proc Natl Acad Sci U S A.

[33] Stuart K, Panigrahi AK, Schnaufer A, Gott JM (2004). Identification and characterization of trypanosome RNA editing complex components.. Methods in Molecular Biology.

[34] Rigaut G, Shevchenko A, Rutz B, Wilm M, Mann M (1999). A generic protein purification method for protein complex characterization and proteome exploration.. Nat Biotechnol.

[35] Panigrahi AK, Gygi S, Ernst N, Igo RP, Palazzo SS (2001). Association of two novel proteins, *Tb*MP52 and *Tb*MP48, with the *Trypanosoma brucei* RNA Editing Complex.. Mol Cell Biol.

[36] Kable ML, Seiwert SD, Heidmann S, Stuart K (1996). RNA editing: a mechanism for gRNA-specified uridylate insertion into precursor mRNA.. Science.

[37] Seiwert SD, Heidmann S, Stuart K (1996). Direct visualization of uridylate deletion in vitro suggests a mechanism for kinetoplastid RNA editing.. Cell.

[38] Cruz-Reyes J, Zhelonkina A, Rusche L, Sollner-Webb B (2001). Trypanosome RNA Editing: Simple Guide RNA Features Enhance U Deletion 100-Fold.. Mol Cell Biol.

[39] Igo RP, Palazzo SS, Burgess MLK, Panigrahi AK, Stuart K (2000). Uridylate addition and RNA ligation contribute to the specificity of kinteoplastid insertion RNA editing.. Mol Cell Biol.

[40] Igo RP, Weston D, Ernst N, Panigrahi AK, Salavati R (2002). Role of uridylate-specific exoribonuclease activity in kinetoplastid RNA editing.. Eukaryot Cell.

[41] Igo RP, Weston DS, Ernst NL, Panigrahi AK, Salavati R (2002). Role of uridylate-specific exoribonuclease activity in *Trypanosoma brucei* RNA editing.. Eukaryotic Cell.

[42] Pfaffl MW (2001). A new mathematical model for relative quantification in real-time RT-PCR.. Nucleic Acids Res.

[43] Ramakers C, Ruijter JM, Deprez RHL, Moorman AFM (2003). Assumption-free analysis of quantitative real-time polymerase chain reaction (PCR) data.. Neurosci Lett.

