# Supplementary figures and images for: KREX2 Is Not Essential for Either Procyclic or Bloodstream Form Trypanosoma brucei
Source: PLoS One. 2012 Mar 15;7(3):e33405. doi: 10.1371/journal.pone.0033405 (PMC3305318; doi:10.1371/journal.pone.0033405)

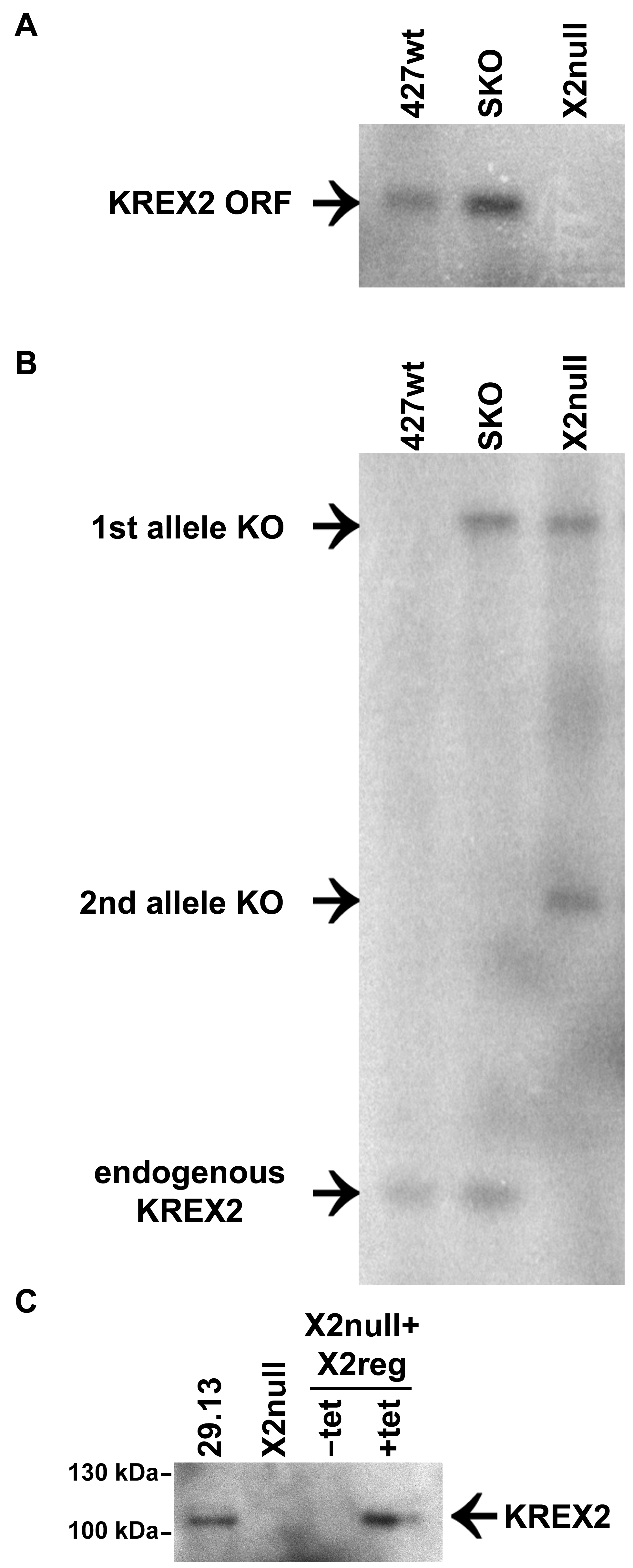

Supplement: Figure S1 — Southern and Western analyses demonstrate elimination of KREX2 in BF-KREX2-null and PF-KREX2-null cells. A. Genomic DNA from parental BF-427wt and derived KREX2 single knockout (SKO) and KREX2-null (X2null) cell lines was subjected to Southern analysis using a radiolabeled probe to detect the KREX2 open reading frame (KREX2 ORF). The band corresponding to KREX2 is present in DNA from either BF-427wt or BF-KREX2-SKO cell lines, but completely absent in DNA from BF-KREX2-null cells. B. The same genomic DNAs used in panel A were also analzyed using a radiolabeled probe to detect the 3′ intergenic region of KREX2. Hybridization with this probe permits simultaneous detection of the endogenous KREX2 alleles, the first allele knockout (1st allele KO) with T7 RNA polymerase and NeoR, and the second allele knockout (2nd allele KO) with tetracycline regulator and HygR. While parental BF-427wt cells have only endogenous KREX2, the BF-KREX2-null cells lack endogenous KREX2 and only display hybridization consistent with the knockout constructs that replaced each KREX2 allele. C. Western analysis of ∼20S peak glycerol gradient fractions from parental PF 29.13 or PF-KREX2-null (X2null) and derived cells. Anti-KREX2 antibody reveals presence of KREX2 in 29.13 cells, and absence in PF-KREX2-null cells. Expression of the tetracycline (tet) regulatable ectopic KREX2 allele in the PF-KREX2-null+KREX2Reg cell line (X2null+X2reg) was demonstrated in the presence of tet (+tet), but not in its absence (−tet). The sizes of proximate marker bands are as indicated. The amount of KREX2 in extracts from BF cells was below the limit of detection with this antibody (data not shown). Southern analysis. Genomic DNA was isolated from each cell line (ACS protocol). For each cell line, 20 µg of genomic DNA was digested with either EcoRI or a combination of BamHI and KpnI and then fractionated by electrophoresis on 0.8% agarose gel. EcoRI digestion of genomic DNA generates a 1141 bp fragment (WT), a 55 [file pone.0033405.s001.tif]
